# Iron Supplementation Therapy, A Friend and Foe of Mycobacterial Infections?

**DOI:** 10.3390/ph12020075

**Published:** 2019-05-17

**Authors:** Rafiou Agoro, Catherine Mura

**Affiliations:** 1 Medical and Molecular Genetics, Indiana University, School of Medicine, Indianapolis, IN 46202, USA; 2 Experimental and Molecular Immunology and Neurogenetics (INEM), Mixed Research Unit UMR7355, National Center for Scientific Research and Orléans University, 45071 Orléans, France

**Keywords:** iron, mycobacteria, immunity

## Abstract

Iron is an essential element that is required for oxygen transfer, redox, and metabolic activities in mammals and bacteria. Mycobacteria, some of the most prevalent infectious agents in the world, require iron as growth factor. Mycobacterial-infected hosts set up a series of defense mechanisms, including systemic iron restriction and cellular iron distribution, whereas mycobacteria have developed sophisticated strategies to acquire iron from their hosts and to protect themselves from iron’s harmful effects. Therefore, it is assumed that host iron and iron-binding proteins, and natural or synthetic chelators would be keys targets to inhibit mycobacterial proliferation and may have a therapeutic potential. Beyond this hypothesis, recent evidence indicates a host protective effect of iron against mycobacterial infections likely through promoting remodeled immune response. In this review, we discuss experimental procedures and clinical observations that highlight the role of the immune response against mycobacteria under various iron availability conditions. In addition, we discuss the clinical relevance of our knowledge regarding host susceptibility to mycobacteria in the context of iron availability and suggest future directions for research on the relationship between host iron and the immune response and the use of iron as a therapeutic agent.

## 1. General Context

Mycobacterial infections in human populations are increasing worldwide and remain a major cause of morbidity and mortality, making them a major public health concern. The emergence and spread of multi-drug resistant (MDR) and extensively drug-resistant (XDR) mycobacterial strains have shifted our interest to explore new therapeutic approaches. Iron plays a crucial role in the pathophysiology of mycobacteria and should be of great interest for future therapeutic strategies. In this context, the development of an effective iron-based therapy requires a detailed understanding of the role of iron during mycobacterial infections as a target involved in the resistance and/or susceptibility of host. 

## 2. Mycobacteria: The Smart Pathogens

**Figure 1:**
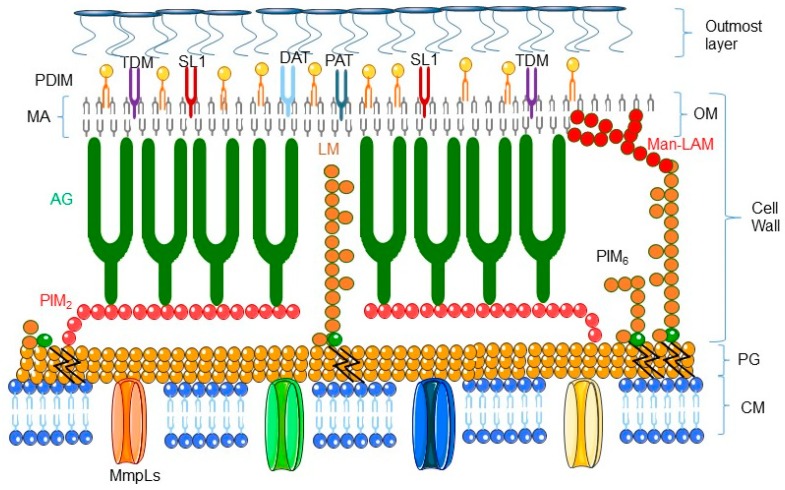
Schematic representation of the cell envelope of *Mycobacterium tuberculosis* (adapted from [2]). The cell envelope of *M. tuberculosis* includes an inner cell membrane (CM), a network of peptidoglycan (PG) with covalently attached macromolecules: AG, PIM_2_, PIM_6_, LM, and Man-LAM; an outer membrane (OM) composed of mycolic acid (MA), attached to TDM, DAT, PAT, PDIM, and SL-1; and an outmost layer of polysaccharides and proteins. AG: arabinogalactan; DAT: diacyltrehalose; LM: lipomannan; Man-LAM: mannose-capped lipoarabinomannan; PAT: poliacyltrehalose; PDIM: phthiocerol dimycocerosate; PIM_2_: phospho-*myo*-inositol-dimannoside; PIM_6_: phospho-*myo*-inositol-hexamannoside; MmpL: mycobacterial membrane protein large; TDM: trehalose dimycolate; SL-1: sulphoglycolipid.

The genus *Mycobacterium* comprises more than 150 species that reside in a wide variety of habitats [1]. These bacteria are typically characterized by the structure of their cell envelope (see Figure 1 representing the structure of mycobacterial envelope and for review [2]). Mycobacteria are divided into three groups, the *Mycobacterium tuberculosis* complex (*M. tuberculosis*, *M. africanum, M. bovis*, *M. canetti,* and *M. microti*), lepromatous mycobacteria (*Mycobacterium leprae*), and non-tuberculosis mycobacteria (NTM), a group which contains only a few pathogenic species involved mostly in lung infection in immunocompromised individuals [3,4]. 

Host immune systems have evolved antimicrobial strategies, whereas mycobacteria which are facultative intracellular pathogens have developed new ways to survive in previously inaccessible niches and hostile environment. The *Mycobacterium tuberculosis* complex species are mainly restricted to macrophages as host cells and induce a chronic immunopathology. Macrophages internalize mycobacteria by phagocytosis, but only pathogenic mycobacterial species can survive and proliferate inside these cells. In hosts cells, mycobacteria are located in the phagosome and thus exposed to hostile conditions, such as oxidative stress, hypoxia, and nutrient restriction also known as nutritional immunity. 

The pathogenic mycobacteria have adapted to the hostile environment of the phagosome by entering into a dormant state. Indeed, during chronic infection, *M. tuberculosis* survives nutrient starvation by using the β-oxidation pathway, which allows fatty acids to be utilized as a unique carbon source. Dormant *M. tuberculosis* cells are characterized by a distinct cell wall lipid content. Indeed the β-oxidation pathway is a source of phthiocerol dimycocerosates (PDIM), glycolipids diacyltrehalose (DAT), and polyacyltrehalose (PAT), and sulfolipid (SL-1) [2]. To establish residence in macrophages, the pathogenic mycobacteria slow down their growth and can lay in a ‘dormant’ state for decades, further limiting the risk of destroying their host macrophages and allowing them to survive harsh living conditions. 

### 2.1. Host Response to Mycobacteria Through Immune Cell Activation 

The host sets up a complex immune response involving both innate and adaptive components that are specific to persistent intracellular pathogens. The host often sequesters mycobacteria in an organized structure named granuloma, composed of center of phagocytic cells surrounded by T and B lymphocytes, that regulates the immune-mediated containment of the infection. 

Granulomas are hypoxic and nutrient-restricted hostile environments for mycobacteria that contain the infection [5]. In most cases, granulomas do not develop into an active site of infection and can resolve infection or support bacterial survival over long period of time. The resident macrophages phagocytize and eradicate most infecting mycobacteria, preventing the mycobacteria colonization and further mycobacterial infection. Indeed, activated macrophages can induce bacteriostatic and bactericide effect on mycobacteria, but mycobacteria can reside within nondegradative macrophages. Once infected, only 5–10% of immunocompetent humans exposed to *M. tuberculosis* develop disease. For a successful infection, mycobacteria must not only avoid destruction and survive within phagosomes but must also escape from the initially infected resident macrophage to spread to other areas and infect growth-permissive monocytes to cause disease. This reactivation process depends on the presence of metabolically active bacilli. *M. tuberculosis* infection can manifest as acute or chronic disease or be clinically asymptomatic with the potential to emerge from a latent form to an active form of infection [6]. Phagocytic cells are key biological element involved in the control of mycobacterial infection, although they also assist in their subsequent dissemination.

### 2.2. Host Response to Mycobacteria Through Oxidative Stress Induction: A Critical Role of Iron

Once stimulated, upon mycobacteria infection, TLRs promote the innate immune response and the production of microbicidal effectors, such as reactive nitrogen intermediates (RNI) and reactive oxygen species (ROS) produced by phagolysosomes from macrophages, and microbicidal peptides such as lactoferricin and defensins [7]. 

Accumulating evidence suggests an important role of macrophage-derived nitric oxide (NO) in protecting host cells against intracellular pathogens. Nitric oxide (NO) is a chemical mediator and regulator that has physiological and pathophysiological roles in mammals as well as a significant role in inflammation. NO is produced by the nitric oxide synthase (NOS) enzyme family, which catalyzes the two-step conversion of L-arginine to L-citrulline and NO. The inducible nitric oxide synthase (iNOS or NOS2), a form of NOS is transcriptionally induced in IFNγ-activated macrophages, inducing high production of NO [8]. NO possesses cytotoxic properties through interactions of reactive free NO radicals with iron containing enzymes or molecular oxygen and superoxide anion to produce reactive nitrogen species that cause massive oxidative injuries. NO is a powerful reactive molecule that plays a major role in controlling mycobacterial infections. Indeed, mice deficient in NOS2 activity are highly susceptible to acute and chronic *M. tuberculosis* infection compared to wild-type mice [9,10]. 

**Figure 2:**
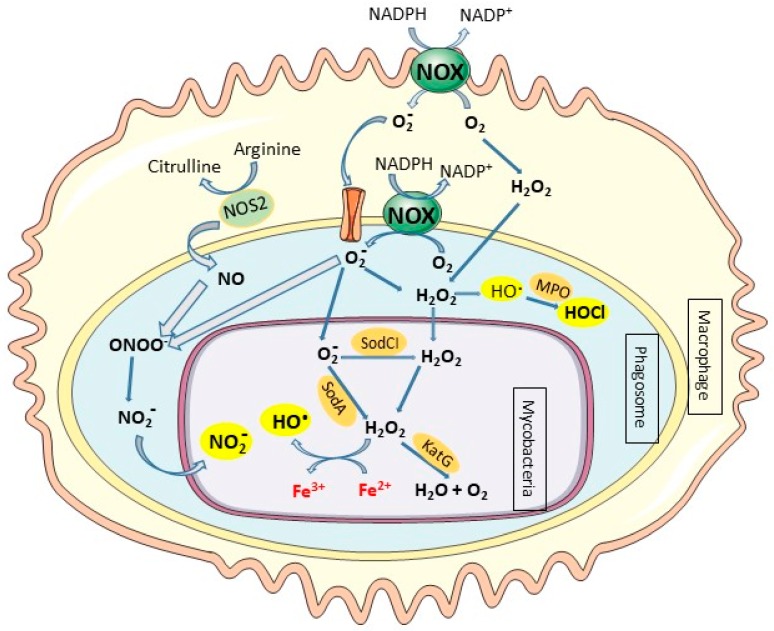
Series of reactive oxygen species generated in response to intracellular mycobacteria infection. Macrophages and neutrophils produce reactive oxygen species to kill intracellular microbes in a series of reaction which can be initiated by the conversion of NADPH to NADP ^+^ via the NADPH oxidase (NOX) and the production of superoxide O_2_^−^ in presence of O_2_. O_2_^−^ can enter in the phagosome and later in the mycobacteria through porins or crosses the outer membrane that is partially permeabilized by antimicrobial peptides. O_2_^−^ is a substrate for superoxide dismutase (SodCI, SodA) to produce H_2_O_2_ which can freely diffuse across membranes. The hydroxyl radical (OH^•^) is produced from H_2_O_2_ and superoxide via Fenton reaction. Mycobacteria produce the catalase KatG to detoxify H_2_O_2_. The production of NO from arginine metabolism via nitric oxide synthase (NOS2) can generate nitric oxide which in presence of O_2_^−^ produce ONOO^−^ and NO_2_^−^, which present some toxic proprieties against mycobacteria.

Phagocytes can eliminate invading pathogens via oxygen-dependent and oxygen-independent mechanisms. The activated phagocytes induce the NADPH oxidase system, a membrane-bound complex located in the plasma membrane and in the phagosomal membranes of phagocytic cells that reduces O_2_ to superoxide anion (O_2_^•−^) and triggers oxidative bursts [11]. Superoxide anion (O_2_^•−^) spontaneously converts to hydrogen peroxide (H_2_O_2_), and H_2_O_2_ can further undergo a Fenton reaction in the presence of iron to yield highly reactive hydroxyl radicals (OH^•^) (Figure 2) [12]. The presence of O_2_^•−^ and NO will further generate peroxynitrite (ONOO^−^/ONOOH), the most injurious reactive nitrogen species (RNS), and other reactive free radicals (Figure 2) [13,14]. These molecules can damage a variety of biomolecules, including DNA, which effectively kills the pathogen and inhibits its dissemination [15]. Neutrophils are professional phagocytes that play protective roles in mycobacterial infections through the release of preformed oxidants and proteolytic enzymes which are discharged during the degranulation process [16,17]. Neutrophils contain cytoplasmic granules that contain large amount of myeloperoxidase (MPO) that uses H_2_O_2_ to catalyze the production of highly toxic ROS like hypochlorous acid (HOCl) in the presence of anion chloride (Cl^−^). The production of ROS, O_2_^−^, H_2_O_2_, and HOCl by phagocytic cells in response to infection is a highly effective microbicidal mechanism that is also referred to as a respiratory burst. In contrast to neutrophils, mature macrophages contain much less concentrations of MPO and thus are unable to kill pathogenic intracellular microorganisms by this system [18].

In humans, genetic deficiency for one of the subunits of NADPH oxidase causes an inherited immunodeficiency, chronic granulomatous disease (CGD), which is characterized by dysregulated inflammation and recurrent infections with catalase-positive microorganisms, including *M. tuberculosis* and *M. bovis* BCG [19]. Moreover, subjects with MPO deficiency have an increased susceptibility to infection [20]. However, it has been demonstrated that MPO-loaded macrophages still ingest *M. tuberculosis* and do not show a significant mycobactericidal activity despite a highly susceptibility of mycobacteria to the system in vitro. This failure of peroxidase-loaded macrophages to kill *M. tuberculosis* may result from the presence of efficient detoxifying mechanisms in the mycobacteria [18]. The lack of granular MPO in mature macrophages may explain the tendency of mycobacteria to infect these cells that favor the survival and proliferation of pathogenic intracellular microorganisms. 

## 3. The Iron Tug-of-War Between Host and Mycobacteria

### 3.1. The Importance of Iron for Mycobacteria

Iron is a crucial nutrient for mycobacteria, as it represents an essential structural and catalytic cofactor for many metabolic enzymes. In mycobacteria, iron is used as cofactor of enzymes involved in amino acid and nucleic acid biogenesis, such as those with pyrimidine synthesis and ribonucleotide reductase activities, as well as enzymes in the tricarboxylic acid cycle, superoxide dismutase, 3-deoxy-D-arabino-heptulosonate 7-phosphate synthase, and proteins participating in electron transport. Specifically, in *M. tuberculosis,* iron is an obligate cofactor for at least 40 different enzymes encoded in its genome [21]. 

In mycobacterial host mammals, iron is present in the cytoplasm of cells at a very low level in a ferrous soluble, chelatable state that constitutes the labile iron pool. The labile iron pool is potentially toxic as it can generate reactive oxygen species. Thus, most of the iron is sequestered in complexes with iron binding proteins. In blood, the free iron concentration is approximately between 10^−18^ and 10^−12^ µM and that of total serum iron is 10–50 µM and most iron circulates bound to transferrin glycoprotein or to lactoferrin [22]. Macrophages are characterized by high iron flux due to recycling of iron from senescent erythrocytes and the internalization of iron via specific cell surface receptors for transferrin, lactoferrin, and hemoglobin-haptoglobin [23], and thus represent a favorable niche for mycobacteria to acquire iron.

Granulomas are microenvironments in which mycobacteria brave starvation including iron deprivation. However, virulent mycobacteria are capable of long-term persistence without growth. Granulomas formation in response to *M. tuberculosis* infection are heterogeneous mainly characterized by solid cellular granulomas, cavitary granulomas and necrotic caseous granulomas in advanced tuberculosis infection [24]. Transcriptional analysis has indicated that solid cellular granulomas express high levels of iron uptake genes such as heme binding proteins, hemoglobin receptor, and transferrin receptor 1 encoded by *TFRC*; the cavity granulomas express high ferritin and heme oxygenase suggesting a permissive iron environment. Besides, necrotic and cavity granulomas exhibit gene expression of extracellular of iron, hemoglobin and heme sequesters such as transferrin, haptoglobin, and hemopexin, in addition to lactoferrin, lipocalin and calprotectin indicating host iron restriction [25]. 

In a prolonged iron starvation environment such as granulomas, *M. tuberculosis* stops the replication process but remains metabolically active, with intact cell envelope, high expression of iron acquisition *mbt* genes, reduced heme and iron-proteins synthesis and repressed oxidative phosphorylation [25]. Like all successful pathogens, mycobacteria have developed sophisticated mechanisms to compete with host iron-scavenger proteins for iron acquisition, to transport and store iron and to acquire iron from both extracellular transferrin, lactoferrin and intracellular iron pools [26].

#### 3.1.1. Siderophores: The Mycobacteria Iron Scavengers 

**Figure 3:**
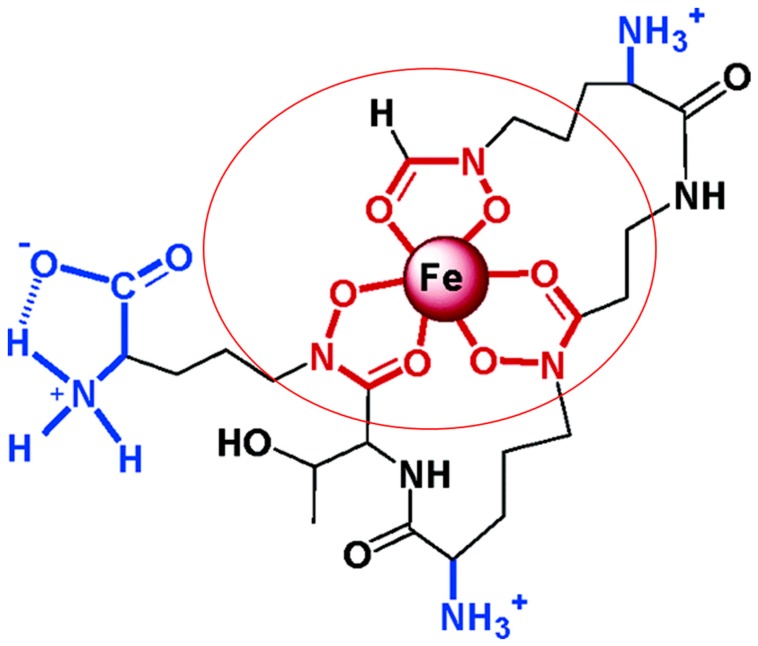
Structure of exochelin siderophore of *M. smegmatis* with its iron chelation proprieties. The iron-chelating residues of exochelin with their interaction with iron molecule is shown in red circle (adapted from [29]).

In iron-deficient environments, mycobacteria produce small iron-binding molecules called siderophores. These molecules have a high affinity for iron and scavenge metal ions from host insoluble and protein-bound iron. Mycobacterial siderophores can be divided into siderophores from non-pathogenic and pathogenic mycobacteria. Exochelins are extracellular and hydrophilic peptidic siderophores utilized mainly by non-pathogenic mycobacteria, such as *M. smegmatis* and *M. neoaurum*. *M. leprae,* a pathogenic mycobacterium also utilizes the siderophore exochelin for iron acquisition [27,28]. A structure of *M. smegmatis* exochelin siderophore showing its iron chelation abilities is represented in Figure 3 [29].

**Figure 4:**
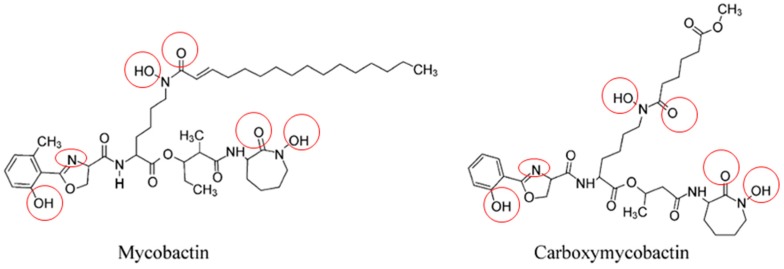
Structure of mycobactin and carboxymycobactin siderophores of pathogenic mycobacteria. This figure represents mycobactin and carboxymycobactin from *M. tuberculosis* with the iron-chelating residues circled in red.

**Figure 5:**
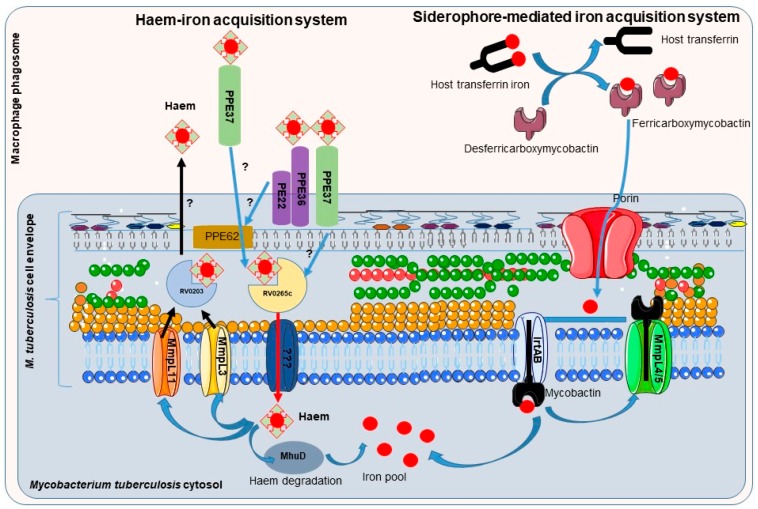
Heme-iron and siderophore-mediated iron acquisition of *Mycobacterium tuberculosis* in macrophage phagosome (adapted from [55]). *M. tuberculosis* has both heme and non-heme iron uptake pathways. *M. tuberculosis* secretes and express on its outer membrane PPE36/37 which can bind to heme and induce heme importation across the outer membrane. Then heme is linked to Rv0265c which facilitate heme transport to the cytosol using an unknown heme importer. In the cytosol, heme is degraded by the heme-degrading protein MhuD and iron is extracted from heme. It is also possible than heme can also be exported from cytosol through MmpL3/11 transporters and then linked to Rv0203 and later exported outside the bacteria. *M. tuberculosis* can also acquire iron through the siderophore-mediated iron acquisition system. *M. tuberculosis* desferricarboxymycobactin competes with host iron transferrin to acquire iron in phagosome. Iron is then bound to mycobactin which transports iron across the inner membrane through the IrtAB transporter complex. Once in the cytosol, iron is dissociated from mycobactin which is then exported from the cytosol through MmpS4/L4 and MmpS5/L5 complex.

Mycobactins are derived from salicylic acid and include the mycobactin and carboxymycobactin forms of siderophores isolated from pathogenic mycobacteria (Figure 4), such as *M. tuberculosis, M. bovis, M. bovis* BCG, *M. africanum* and *M. microti*. Mycobactin is lipophilic and envelope-associated while carboxymycobactin is an extracellular hydrophilic molecule [30,31]. Desferricarboxymycobactin competes with host iron-binding proteins for iron; it chelates Fe^3+^ iron bound to host transferrin after phagosome fusion with early endosomes, as well as from lactoferrin and ferritin. In early endosomal phagosomes, mycobacteria communicate with the endocytic iron uptake system of the host macrophage and take advantage of this source of iron (Figure 5) [32,33]. Iron-siderophore complexes ferricarboxymycobactins are transported through the Msp (*Mycobacterium smegmatis* porin) family porins, a multisubunit transport system of the mycobacterial outer membrane. Subsequently, ferricarboxymycobactin transfers iron to the cell-wall associated mycobactin or delivers it to the inner membrane-bound iron-regulated transporter A and B (IrtAB). IrtAB, an ATP-binding cassette transporter synthesized in iron limited condition, mediates the reduction of the iron from internalized ferric-siderophore complexes into Fe^2+^ and its release [31,34,35,36,37]. The export and recycling of desferricarboxymycobactin through the inner membrane is carried out by the MmpS4/MmpL4-MmpS5/MmpL5 transporter complex formed with mycobacterial membrane proteins (Mmps). The recycling of desferricarboxymycobactin is critical for bacterial survival itself [38] (Figure 5). Indeed, genetic disruption of the recycling process induces the accumulation of these molecules in mycobacteria and poisons *M. tuberculosis* [38,39]. 

Mycobactins are produced by nonribosomal peptide synthetase enzyme system and requires multiple enzymes encoded in two gene clusters *mbt*A-J and *mbt*K-N in *M. tuberculosis* [40,41]. Genetic disruption of siderophores expression impairs the growth of *M. tuberculosis* in mice and macrophages, demonstrating the essential role of iron acquisition for mycobacteria virulence [42]. The pathogenicity of bacteria depends on secretion systems for an efficient transport of biomolecules known as virulence factors. The secretion systems ESX, or Type VII systems, are specific to mycobacteria which required specialized mechanisms for protein transport across the lipid-rich outer membrane barrier. The genes encoding the ESX-1 system have been identified in the genome of the virulent *M. tuberculosis* strain H37Rv, but these genes are absent from the genome of *M. bovis* BCG, which corresponds to the genomic region of difference 1 (RD1) [43]. The ESX-3 system is crucial for iron acquisition in *M. tuberculosis* and *M. smegmatis* contributing to growth and virulence. The esx-3 mutants display severe growth defects in the presence of low concentrations of iron, which can be rescued by the addition of iron or heme [44,45]. Indeed, *esx-3* is expressed in response to iron deficiency, and mycobacterial *esx-3* mutant strains synthesize and accumulate dramatic amounts of mycobactin siderophores but are unable to take up iron and grow poorly. In *M.* *tuberculosis*, the addition of mycobactin has been shown to rescue the esx-3 mutant growth defect [46,47]. Further studies identified secreted PE5-PPE4 proteins encoded by esx-3 as being crucial for iron acquisition, while the virulence phenotype correlates with the secretion of EsxG-EsxH complex that impairs phagosomal maturation [47]. 

#### 3.1.2. Mycobacteria Heme-Iron Acquisition Systems 

Mycobacteria residing in macrophage phagosomes can also acquire heme as an iron source after the phagocytosis of senescent red blood cells by macrophages, a process termed erythrophagocytosis. Indeed, the attenuated growth of *M. tuberculosis* in low-iron medium or mycobactin-deficient mutant can be rescued by heme supplementation [48]. Extracellular heme can be recovered by mycobacteria through the secretion of hemolysin whose encoding gene (*tlyA*) has been identified in several strain of mycobacteria (*M. tuberculosis*, *M. bovis* BCG, *M. leprae*, *M. avium*) [49]. Further, the heme-binding protein Rv0203 [35,50] and membrane heme import proteins from mycobacterial membrane proteins large family, MmpL3 and MmpL11 [50,51] might be involved in heme efflux to protect mycobacteria from the toxicity of excess heme [52]. PPE37 (also named Rv2123), member of the proline–proline–glutamic acid gene family restricted to virulent mycobacterial species, has been recently described as critical for heme-iron acquisition [53]. *Ppe37* gene deletion in *M. tuberculosis* severely attenuates heme-iron acquisition and abrogates growth in a medium with hemin as the sole iron source [53,54]. PPE37 has been identified as a factor of virulence; *M. bovis* BCG has been described recently as severely deficient in heme-iron acquisition ability due to the lack of PPE37; importantly, *M. bovis* BCG exhibits heme iron acquisition as efficient as that of *M. tuberculosis* when complemented with *M. tuberculosis* *PPE37* [53]. Once in mycobacteria cytosol, the heme degrading protein MhuD releases the iron from heme (Figure 5) [51]. 

### 3.2. Mycobacteria Protection against the Harmful Effects of Iron, ROS and RNS

For efficient infection and persistence, pathogenic bacteria have evolved to adapt their metabolism to the hostile environment of the phagosome to survive harmful antimicrobial host defense mechanisms such as iron limitation and the production of toxic reactive oxygen and nitrogen species. Iron is an essential structural and catalytic cofactor for many metabolic enzymes but is also a harmful component due to its ability to generate spontaneously ROS through the Fenton reaction. Therefore, bacterial intracellular iron homeostasis is closely linked to the response to oxidative stresses.

#### 3.2.1. Protection against Excess of Iron

Once captured, iron can be utilized for various metabolic processes while specific proteins are synthesized to store iron and avoid its toxic effects. *M. tuberculosis* encodes two iron storage proteins, BfrA, a bacterioferritin, and BfrB, a ferritin-like protein [56]. Both proteins aggregate to form macromolecular structures consisting of 24 subunits that can hold 600 to 2400 iron atoms per molecule [41]. BfrA is required for the efficient release of stored iron under low iron conditions, while BfrB has a high capacity for iron storage and is a major defense protein under excessive iron conditions [57]. *BfrB* deleted mutant showed drastic loss of viability during iron depletion indicating that iron stored in ferritin is essential for *M. tuberculosis* to survive in iron depleted environment [25]. Several investigations using *M. tuberculosis bfrA* and *bfrB* mutant have demonstrated that these genes are crucial for the ability of mycobacteria to grow and withstand oxidative stress in vitro, as this mutant exhibits a marked reduction in survival inside human macrophages and is unable to establish successful infection in guinea pigs [56,58]. Therefore, although iron is an essential nutrient for mycobacterial growth, iron excess can be detrimental to these pathogens. 

In mycobacteria, intracellular iron levels are regulated by controlling the transcription of genes involved in iron uptake, transport and storage through the iron-dependent transcription factors IdeR, a negative regulator and HupB, a positive regulator [59,60] Under high iron conditions in *M. tuberculosis*, IdeR binds Fe^2+^ and represses the transcription of siderophore synthesis by binding to the IdeR box in promoter of *mbt* genes and iron transport genes as well as *ppe37* gene while inducing the transcription of the genes encoding iron storage BfrA and BfrB proteins [41,59,60]. The disruption of the *ideR* gene in mycobacteria is lethal for these pathogens [59]. A conditional *M. tuberculosis ideR* mutant that is unresponsive to iron under non-permissive conditions is unable to repress iron acquisition and displays attenuated growth in vitro and in vivo, whereas increased cellular iron levels are associated with a high sensitivity to NO and H_2_O_2_ [61]. These results indicate that iron accumulation in mycobacteria exacerbates oxidative stress, providing an additional link between iron homeostasis and virulence in *M. tuberculosis* infection. A recent study has identified several inhibitors of IdeR which interestingly attenuate *M. tuberculosis* growth in vitro [62].

Furthermore, the HupB DNA-binding histone-like protein, which is repressed by IdeR-Fe^2+^ complex under increased iron levels, exerts a positive regulatory role on the expression of mycobactins by binding to the *mbt* promoter upon iron limitation [41,61]. The HupB protein is essential for siderophore synthesis and deficient *hup*B (or *Rv2986c*) fails to grow inside the macrophages and in an axenic culture [61]. HupB plays a critical role in mycobacterial growth especially during exponential growth. Besides its role as a positive regulator of iron acquisition, HupB is involved also in several other biological functions such as cell wall assembly and immunoproliferation in *M. tuberculosis* [41].

#### 3.2.2. Antioxidant Systems in Mycobacteria 

The success of *M. tuberculosis* as pathogen is dependent on its ability to adapt to the harsh environmental and stressful conditions in granulomas. Granulomas impose multiple stress factors, such as NO, CO, and low O_2_, and these changes in redox balance cause *M. tuberculosis* to shift from aerobic to anaerobic metabolic pathways, leading cells to transition from an actively growing state to a dormant state. Mycobacteria express redox sensors during infection, allowing cells to sustain a redox balance that neutralizes the toxic effect of ROS and plays a role in mycobacterial virulence. The DosR/S/T system and the WhiB Fe-S cluster family of proteins are the two primary dormancy-induced signaling pathways in mycobacteria. 

**Figure 6:**
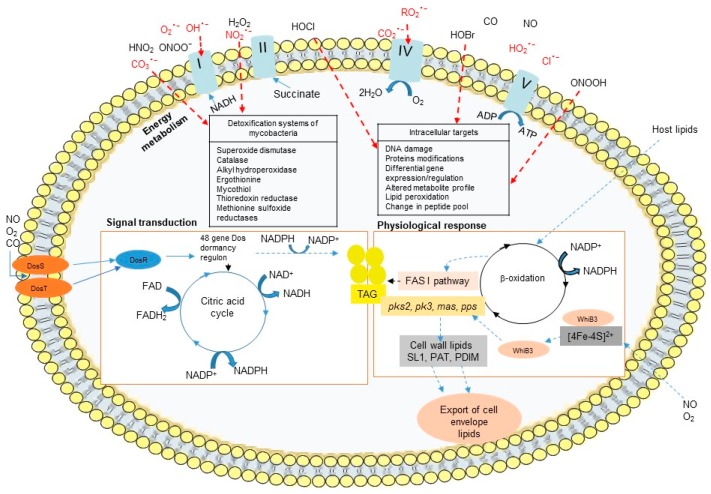
Mycobacterial stress sensors (adapted from [75]) Mammalian host cell generates radicals and gases as a mechanism to counter *mycobacterial* infection. *M. tuberculosis* senses these potentially damaging stresses and responds by adjusting its energy metabolism, physiology and signaling and counters the damage by detoxification processes. *M. tuberculosis* possesses sensing mechanisms such as the Dos dormancy regulon and the WhiB3 redox sensor detecting environmental gases and alterations in its intracellular redox state. The Dos regulon senses O_2_, NO, and CO through the DosS and DosT heme proteins which transduce the signal to DosR leading to the induction of 48-member Dos dormancy regulon. WhiB3 functions as a regulator of cellular metabolism, which responds to O_2_ and NO through its Fe–S cluster and integrates it with intermediary metabolic pathways. WhiB3 is an intracellular redox regulator that dissipates reductive stress generated by utilization of host fatty acids through β-oxidation.

The DosR/S/T dormancy regulon is widely distributed among mycobacterial genomes excepted *M. leprae* which lacks DosR. This Dos regulon, essential for the long-term survival of mycobacteria includes a response regulator (DosR) and two heme iron-containing sensor kinases (DosS and DosT), which control roughly 50 genes that are essential for the establishment and maintenance of a dormant anaerobic state. The Dos regulon responds to diatomic gases (NO, CO, and O_2_). The activation of the DosR transcription factor is modulated by the DosS and DosT heme containing kinases that are susceptible to the cellular redox state and the oxygen levels (Figure 6) [63]. In the presence of oxygen, the heme iron in DosS and DosT are in the ferric form and are inactive, whereas iron deoxidization or NO/CO binding promote their kinase activities [63]. Although, iron deprivation of *M. tuberculosis* does not induce the dormancy regulon and *dosR* mutant survive to iron starvation [25].

In addition to the DosR/S/T system, *M. tuberculosis* senses redox signals such as O_2_ and NO via the WhiB family of iron-sulfur (Fe-S) cluster-containing transcription factors. The *whiB*-like genes are exclusive to actinomycetes, such as *Mycobacterium* and *Streptomyces* spp. *M. tuberculosis* genome contains seven *whiB*-like genes (*whiB1* to *whiB7*). *WhiB3* has been described to contribute to the persistence and virulence of *M. tuberculosis*. The WhiB3 Fe-S cluster senses changes in the intracellular redox environment associated with hypoxia and regulates the metabolic switch for the use of fatty acids as a carbon source, modulating the biosynthesis of the virulence-associated lipids PAT/DAT, SL-1, PDIM, and lipid inclusion bodies (triacylglycerol TAG) (Figure 6). *WhiB3* gene expression is increased in mycobacteria residing in macrophages, enabling them to alleviate the potential harmful effects of redox imbalance [64]. *WhiB3* expression increases in vitro under conditions resembling the phagosomal environment that act as signal for mycobacterial dormancy, such as acid-stress medium, oxidant medium, or low-nutrient concentrations, while it decreases under low iron conditions [65]. WhiB3 binds NO and is degraded by O_2,_ and a *M. tuberculosis whiB3*-deletion mutant severely impairs growth on nutrient-depleted medium that cannot be rescued by nutrient supplementation [66]. Furthermore, WhiB3 allows mycobacteria to adapt to low oxygen tensions through transcriptional regulatory networks that maintain redox homeostasis.

Besides WhiB3, others WhiBs functions have been described in mycobacteria. WhiB4 is highly sensitive to oxygen exposure regulating antioxidant systems as well as being required for the virulence-associated formation of well-organized granulomas during *M. marinum* infection [67]. The WhiB5 transcriptional regulator controls the expression of genes involved in *M. tuberculosis* reactivation and aerobic/anaerobic metabolism that modulate the levels of mycobacterial infection, granuloma formation and dissemination [68]. The WhiB6 regulator reacts with NO and regulates the expression of genes associated with the ESX-1 secretion system and Dos dormancy genes, modulating the virulence and granuloma formation as well as replication and dissemination of *M. marinum* [69]. 

In addition, mycobacteria express several oxidative response gene products that degrade microbicidal oxidative metabolites and are crucial for mycobacterial survival in macrophages. *M. tuberculosis* synthesizes the superoxide dismutase SodC that converts O_2_^−^ to O_2_ and H_2_O_2_ as well as the catalase-peroxidase KatG to degrade H_2_O_2_ [70,71,72,73]. Under high iron conditions, *M. tuberculosis* upregulates the expression of *katG, bfrA*, and *bfrB* indicating the importance of increasing iron storage capacity and preventing oxidative damage that may be caused by excess iron. Other molecules such as redox enzymes such as thioredoxin reductase (TPx), alkyl hydroperoxidase (AhpC), and methionine sulfoxide reductases (MSRs), mycothiol and ergothioneine produced by mycobacteria, and lipoarabinomannan (LAM) a cell wall component and potent oxygen radical scavenger in *M. tuberculosis* and *M. leprae* species are also major virulence factors [74].

## 4. Implication of Iron in the Host Arsenal Defense against Mycobacterial Infection 

Mammals have developed a series of strategies to fight pathogens by limiting nutrient access, such as iron and zinc, as part of a nutritional immunity process. Limiting the delivery of essential elements to microbes is a strategy to starve invading pathogens and inhibit pathogen growth and subsequently disease progression [76]. In mammals, the levels of circulating iron and zinc rapidly decrease with inflammation and during acute phase of infection, establishing the hypoferremia and hypozincemia conditions in consequence of the expression of acute phase proteins such as cytokines, cellular proteins, and hormones. The increase of acute phase protein restricts iron and zinc availability suppressing then pathogenic multiplication. 

### 4.1. Host Cellular Iron Metabolism during Mycobacteria Infection 

The host immune response regulates the expression of a series of mRNAs including iron metabolism proteins that have antimicrobial properties by scavenging iron. Iron within macrophages is limited through the activity of the immunomodulatory cytokine, interferon gamma (IFNγ). IFNγ-stimulated monocytes have been shown to inhibit *Legionella pneumophila* multiplication, which is neutralized by holo-transferrin, a source of iron [77]. In addition, lactoferrin, an abundant neutrophil-derived protein released upon activation at sites of inflammation, possesses a greater iron-binding affinity than transferrin and has a broad mycobacteriostatic activity, by sequestering iron; moreover, congenital lactoferrin deficiency leads to recurring infection [78,79]. Thus, during inflammation, the down-regulation of the transferrin receptor as well as the increase in lactoferrin iron-binding protein levels, deplete iron from activated macrophages. This decrease of iron uptake can be related in some studies by a decrease of intracellular iron-storage protein ferritin, although other investigations using *Mycobacterium avium* have reported an induction of ferritin expression in macrophages by activating toll-like receptor 2 [80].

In macrophages phagosomes, mycobacterial metabolism is influenced by the iron uptake system of the host early endosomal system. Thus, the increase in iron-binding proteins in the host during the immune response restricts iron bioavailability for intracellular pathogens. However, high iron levels in addition to ferritin and lactoferrin have been noticed in *M. paratuberculosis* infected mononuclear phagocytic cells compared to low levels observed in non-infected cells [81]. 

It is likely that siderophores efficiently capture iron from the host iron-binding proteins and provide a source of iron to mycobacteria, promoting their intracellular growth. Recently, it has been shown that mice lacking ferritin H in the myeloid-derived cell population rapidly succumb to *M. tuberculosis* infection. This phenomenon is associated with an alteration of iron homeostasis with an increased ferroportin expression in lung. Moreover, these cell-specific knockout mice exhibit, a strong Th-1 immune response upon infection with an upregulation of Arginase 1, an enzyme involved in the conversion of arginine to ornithine thus depleting the Arginine substrate for the enzyme iNOS to synthetize NO [82]. Thus, these observations highlight a role of iron distribution in the immune response against mycobacterial infection.

Natural resistance-associated macrophage protein 1 (Nramp1 or Solute carrier family 11A1) was the first host genetic locus shown to confer resistance to several intracellular microorganisms. The *Slc11a1* gene encodes a protein primarily expressed in macrophages located within the late endocytic compartment and that is recruited to the membrane of the phagosome upon phagocytosis [83]. Early during infection, inbred mice strains can be separated as being resistant (DBA/2, C3H, CBA, and C.D2) or susceptible (C57BL/6 and BALB/c) to mycobacterial infection; these two different group of mouse strains have showed differential capacities of host macrophages to neutralize phagocytized mycobacteria and to control microbial replication in host cells [84,85,86]. Susceptible mouse strains infected with *M. avium* develop a more severe anemia (BALB/c) compared to a resistant mouse strains (C.D2) [87]. Slc11a1 transports divalent metals such as iron and manganese across phagolysosomal membrane and the susceptibility is caused by a single substitution in *Slc11a1* conferring a null allele. Similarly, the *Slc11a1*-deficient mouse strain showed an uncontrolled early phase of T cell-mediated immunity with high intracellular bacterial replication, while the late phase was preserved [88,89,90]. 

The Slc11a1 metal transporter plays a role in the virulence of mycobacteria, however its function is still controversial. Slc11a1 could either transport metals into the phagosome and exert a protective effect against intracellular mycobacteria by triggering the production of NO and hydroxyl radicals and the acidification of the microbe-containing phagosome, or Slc11a1 could transport metals out of the phagosome, thus limiting the availability of essential metals [91]. In humans, a differential susceptibility to mycobacterial infections, such as *M. tuberculosis,* is associated with *SLC11A1* polymorphisms and has been observed in a high-incidence community in South Africa [92,93,94].

### 4.2. Systemic Iron Homeostasis during Mycobacterial Infections

Hepcidin peptide, produced mainly by hepatocytes, is a master regulator of systemic iron homeostasis [95,96]. Hepcidin binds to ferroportin, the sole known iron exporter, and induces its internalization and degradation [97]. The increase in hepcidin reduces the level of ferroportin, resulting in the inhibition of iron flux to blood circulation. The expression of the *Hamp1* gene that encodes hepcidin is upregulated by increased iron levels through TfR/HFE MHC class I-like protein interactions and the Bmp/Smad pathway. Hepcidin is also upregulated upon inflammation through LPS-induced stimulation and the IL-6 cytokine pathway [95,98] as well as by Smad signaling [95,98,99,100,101,102,103]. Other regulators of hepcidin have been identified, such as erythroferrone and heparin known as negative regulators of hepcidin [104,105,106]. 

In mice, upon inflammation, high hepcidin levels decrease intestinal iron absorption and the release of recycled iron from macrophages, inducing hyposideremia of acute inflammation that are associated with low plasma iron and transferrin saturation [100,107]. Thus, upon inflammation, the increase in hepcidin and lipocalin 2 levels promote hyposideremia and the development of anemia of inflammation upon long-term infection or chronic disease [108]. The upregulation of hepcidin is an attempt to limit iron bioavailability to pathogens and inhibit microbial growth. However, controversial results have been observed regarding the expression of hepcidin during mycobacterial infection. 

Macrophages infection with *M. avium* and *M. tuberculosis*, and mycobacterial components such as Toll-like receptor agonists, have been shown to stimulate hepcidin expression in vitro [109,110]. However, in vivo, the levels of hepcidin mRNA have been observed to remain unchanged in wild-type mice infected with *M. avium* while lipocalin 2 is upregulated. In mice, hepcidin mRNA levels even decreases late post-infection in liver of wild-type mice infected with *M. tuberculosis* [111], while a study of mice infected with *M. bovis* BCG indicated a late increase in hepcidin levels in liver [112]. 

Mycobacterial infection models using mice infected with *M. tuberculosis, M. bovis* BCG and *M. avium* have revealed an increase in the expression of *ferroportin* mRNA [87,111,112]. Moreover, ferroportin overexpression in macrophages which promotes iron export leads to a lower mycobacterial burden, lower iNOS production, and phagocytic ability compared to normal macrophages infected with *M*. *tuberculosis* in vitro [113]. Mice given an iron rich diet and infected with *M. bovis* BCG showed a lack of increase of *ferroportin* and higher *iNOS* expression compared to infected mice under a replete diet [112]. These results suggest that a reduction in intracellular iron levels may interfere with macrophage effector functions, such as NO production. Moreover, a human pilot study demonstrated an association between polymorphisms in the *ferroportin* gene and tuberculosis susceptibility, providing evidence of an interaction between iron distribution and mycobacterial growth [114].

### 4.3. Iron and Antimicrobial Peptides

The toll like receptor 4 agonist, LPS induces inflammation and a broad range of immune mechanisms, including hypoferremia with low serum iron and transferrin saturation. In the late 1960s, Kochan et al showed that the serum from guinea pigs which received a LPS treatment has a tuberculostatic effect in axenic culture [115]. Thus, it is likely that the synthesis of the iron regulatory proteins hepcidin and lactoferrin upon inflammation play a dual role against mycobacterial infection acting as an iron regulatory protein and an antimicrobial peptide. 

Hepcidin was first isolated due to its antimicrobial properties [116,117]. Hepcidin is a peptide that is structurally similar to β-defensin and exhibits a direct bactericidal effect against a broad range of microorganisms, including mycobacteria [116,117]. Hepcidin is expressed at a basal level from hepatocytes and upregulated by high levels of iron in the body; hepcidin expression is also upregulated in hepatocytes and macrophages upon inflammation. Interestingly, hepcidin peptide has been reported to be expressed and localized in mycobacteria-containing phagosomes and is associated with a direct antibacterial activity against *M. tuberculosis* in vitro [109]. More recently, it has been shown that *M. bovis* BCG infection in mice upregulates hepcidin in the liver and in macrophages [112]. The upregulation of hepcidin expression in macrophages may play a local role in iron distribution and as an antimycobacterial defensin. 

Some immune cells such as neutrophils have been described to contain important amounts of antimicrobial peptides containing in granules such as antimicrobial peptides of the β-defensin and cathelicidin families, myeloperoxidase, lactoferrin, and lipocalin 2. The infiltration of neutrophils on the sites of infection at the early stage of infection is critical for host defence against *M. bovis* BCG and *M. tuberculosis* mycobacterial infections due the presence of antimycobacterial peptides in these cells [17]. Importantly, some of neutrophils antimycobacterial peptides have also a role in iron homeostasis. For instance, lipocalin-2 secreted mainly by neutrophils, but also by epithelial cells and macrophages binds to siderophores including mycobacterial mycobactins [118] and neutralize their activity. Lipocalin 2 is a bacteriostatic agent that enhances phagocytic bacterial clearance in macrophages and causes iron restriction to inhibit extracellular bacterial and mycobacterial such as *M. bovis* BCG and *M. tuberculosis* growth; importantly, tissue bacterial loads have been described as severe in lipocalin 2 knock-out mice in comparison to wild type mice [87,118,119,120,121,122]. Moreover, lipocalin 2 promotes neutrophil recruitment, which contributes to innate immunity to *M. tuberculosis*, an activity associated with their antimicrobial properties [17]. 

## 5. Future Therapeutic Use of Iron in Mycobacterial Infections: Iron, a Friend or Foe

Mycobacterial infections are particularly difficult to treat due to their intrinsic impermeability, intracellular localization, and low growth rate. To aid in the fight against mycobacteria, it is of great importance that the crucial steps of infection be targeted including growth and entry into dormancy and reactivation. Indeed, the roles of iron acquisition such as siderophore and hemophore pathways, iron storage such as bacterioferritin expression and iron regulatory systems such as iron-based sensors and redox sensors in the mycobacterial proliferation and virulence may suggest that the modulation of iron levels could strengthen the host defense strategy and be used to develop new drug targets against mycobacteria. 

Because the availability of iron appears to be critical for the growth of mycobacteria, the development of drugs to weaken mycobacterial iron acquisition systems may negatively influence mycobacterial viability and dissemination. However, because iron-deficient conditions upregulate the production of siderophores, this condition would favor mycobacteria dormancy and may be detrimental to eradicating mycobacteria. 

Iron can also influence the activity of immune cells and cell metabolism, such as the generation of ROS/NO toxic oxygen metabolites. Because iron plays dual roles in host defense, the modulation of the iron status may be either beneficial or detrimental. Iron-based defense strategies seem promising but require that all the consequences that may occur during infection and immune response are taken into account. A summary of our current knowledge on the effects of iron on mycobacteria is presented from different aspects of experimental procedure and human analysis.

### 5.1. Investigation of the Effect of Iron on Mycobacterial Growth in Host Cell-Free Culture

Several studies have confirmed the role of iron in mycobacterial growth in host cell-free culture. High iron levels promote mycobacterial growth, while iron restriction yields ambiguous results. Iron supplementation in host cell-free culture media promotes the growth of mycobacteria with higher increase in growth for fast growing mycobacteria such as *M. smegmatis* compared to slow growing mycobacteria such as *M. tuberculosis* and *M. bovis* BCG respectively [112,123,124,125,126]. Human serum containing low iron-saturated transferrin (30%) and low serum iron concentration inhibits the growth of mycobacteria such as *M. bovis* BCG in vitro, whereas mycobacterial growth increases with high transferrin iron saturation, either from mice sera (60%) or guinea pigs (84%) [115,127]. 

Synthetic iron chelators and bacterial siderophores effectively remove iron from culture media and blood, such as the natural siderophore desferrioxamine (DFO) from *Streptomyces pilosus* used to treat secondary iron overload in humans. However, chelating agents are less effective at inhibiting the growth of mycobacteria in host cell-free culture. The natural siderophore DFO, as well as chemical chelators such as N,N’bis (2-hydroxybenzyl)-ethylenediamine-N,N’-diacetic acid (HBED), and the 1-amino-3-(2-bipyridyl)isoquinoline derivative VUF-8514 have showed a modest mycobacteriostatic effect on *M. avium* and *M. tuberculosis* in host cell-free culture [112,124,128], whereas phytosiderophore from the root-exudate of *Tephrosia purpurea* has demonstrated a strong effect on *M. tuberculosis* growth [129]. The efficiency of iron chelators may reflect their efficacy at chelating iron but also the countered effect of the induction of mycobacterial iron-acquisition systems in response to a low iron level, a stress signal which increased the capacity of the mycobacteria to acquire iron. Thus, iron restriction can either be detrimental for mycobacteria growth by starving mycobacteria or beneficial by promoting an efficient iron acquisition. 

Therefore, future iron-based therapeutic approaches should be based on developing new compounds that directly inhibit the enzymes involved in siderophore biosynthesis or involved in the downregulation of siderophore genes (for review [130]) or the alteration of heme-iron acquisition pathway [131] or promoting the induction of endogenous siderophore ligands such as lipocalin 2, which acts as a siderophore antagonist by inhibiting mycobacteria growth.

Strategically, the siderophore-mediated iron uptake pathway of mycobacteria, as well as the heme uptake pathway, could be used as vehicle for the delivery of bactericidal molecules inside mycobacteria. These pathways represent an important opportunity to pass through the cell wall barrier of mycobacteria to import antibiotics to kill bacteria, this strategy is known as “Trojan horse” approach to gain access to mycobacteria. Based on this feature to overcome the cell wall barrier characteristic of mycobacteria, novel drug vectors can be formulated. 

### 5.2. Investigation of the Effect of Iron on Mycobacterial Growth in Macrophages 

Macrophages, the main host-cells of mycobacteria, restrict the growth and virulence of mycobacteria compared to host cell-free culture [126]. Both IFNγ and LPS or a co-treatment activate macrophages resulting in a decrease of mycobacteria growth [132], indicating that phagocytosis and/or cell immune response inhibits mycobacterial multiplication. The differential growth of mycobacteria in cultured macrophages as a function of iron levels has been reported, but controversial results have been observed.

Indeed, several studies have indicated that supplementation of mammal cell culture media with free iron, ferrous sulfate or ferric ammonium citrate increases intracellular iron concentrations and enhances the growth of the *M. paratuberculosis, M. avium, M. bovis,* and *M. tuberculosis* in macrophages [112,126,128,132,133,134]. Similarly, to its effect on host cell-free culture, serum containing apo-transferrin limits the growth of *M. avium* in macrophages, which can be prevented by transferrin depletion or the addition of holo-transferrin (500 µg/mL) or iron (8–80 µg/mL of Fe^2+^) [135]. Other studies have showed that the supplementation of serum-free medium with apo-transferrin (50–500 µg/mL; 1.7 mg/mL) or bovine lactoferrin (2 mg/mL; 0.5 mg/mL) inhibits *M. avium, M. bovis,* and *M. tuberculosis* growth in macrophages [124,132,135]. Furthermore, the addition of the iron chelator lactoferrin increases the mycobacteriostatic effect of IFNγ-activated macrophages, while iron citrate (5 µM) or holo-transferrin (1.7 mg/mL) supplementation abrogates the mycobacteriostatic effect of IFNγ-activated macrophages [128,132]. This result is also supported by a study indicating that iron loading influences macrophage polarization towards M2 phenotype [136]. Thus, multiple host and pathogen immune factors with dynamic interactions during mycobacterial infection could exhibit beneficial or detrimental activities for mycobacterial virulence.

The metabolism of macrophage is differentially influenced with iron level exhibiting different outcome of macrophage response to mycobacteria. A treatment of macrophages cell culture medium with a huge amount of iron (500 µM of FeSO4) increases intracellular *M. tuberculosis* viability by compromising macrophage functions such as superoxide production [123]. In contrast, a moderate concentration of ferric ammonium citrate (10 µM) correlates with the production of reactive oxygen species, an impairment in the uptake of *M. bovis* BCG within macrophages and a decrease of bacterial growth [112]. We and others recently showed that iron influences the M1/M2 balance [136,137,138], which plays an important role in the polarization of the immune response. Furthermore, iron downregulates monocyte responsiveness by reducing IFNγ signaling [139] but also decreases TNFα production and restricts *M. tuberculosis* growth [140]. Therefore, the level of iron in the cell culture medium influences intracellular iron levels and cell activities modulating ROS formation and cellular activities.

Iron chelators display varying inhibitory effects against the growth of mycobacteria and in macrophage host-cells activities. Both DFO and silybin iron chelators exert a mycobacteriostatic effect by reducing the growth and viability of extracellular *M. avium* or virulent *M. tuberculosis*. However, DFO suppresses the effects of excess iron on *M. tuberculosis* growth in macrophage culture, while silybin fails to prevent the effects of excess iron [123,128,133]. Indeed, DFO which does not enter into the cells, acts primarily as an iron chelator and has little influence on intracellular parameters, whereas silybin which enters into the host cells, inhibits the formation of superoxide anion radicals and nitric oxide, acting as an antioxidant and anti-inflammatory compound that could promote the transport of iron into mycobacteria [123,128,133,141]. Therefore, the level of iron plays a major role in the intracellular growth of mycobacteria as a nutrient and in the control of gene expression to alleviate iron restriction, but it also has a prominent role in cell defense activities, likely by promoting subsequent chemical reactions requiring iron. 

Upon infection with *M. avium*, *M. tuberculosis*, and *M. bovis* BCG, macrophages upregulate hepcidin expression [110,112]. Furthermore, IFNγ-activated macrophages slightly upregulate hepcidin expression, and IFNγ and mycobacteria infection synergistically induce high levels of hepcidin expression [110]. In infected macrophages, hepcidin has been localized into the mycobacteria-containing phagosomes [109]. The production of hepcidin could locally affect iron distribution with subsequent elevated macrophage iron levels that may either create an iron-favorable environment for pathogens or impair macrophage cytotoxic activity, although it could also have local antimicrobial activity. Therefore, both roles of hepcidin on macrophage defense activities, and mycobacterial viability should be assessed.

### 5.3. Investigation of the Effect of Iron on Mycobacterial Growth In Vivo 

Iron supplementation promotes mycobacterial growth in cell-free culture but gives controversial results in macrophages. Iron is important for mycobacterial growth as well as for macrophage cell properties. In vivo, a complex relationship between iron levels and immune defense against mycobacteria likely results from additional regulation of iron gene expression and immune cell activities. Host iron levels also influence the expression of protein-associated iron in circulating blood and in tissues. Indeed, iron depletion would decrease the expression of hepcidin and ferritin and increase that of the iron-transport protein transferrin. Conversely, iron supplementation increases the expression of hepcidin and decrease the levels of the iron-transport protein transferrin. Interestingly, the infection-induced-inflammation also controls iron metabolism-related genes through the expression of proteins that naturally control iron distribution by increasing the expression of the hormone hepcidin, the iron-binding protein lactoferrin, and the siderophore-binding protein lipocalin 2 and by decreasing the levels of the iron-transport protein transferrin, all of which contribute to anemia of chronic inflammation to maintain bactericidal conditions. Therefore, the scientific rationale behind the current attempts to use iron as therapeutic agent will require a thorough understanding of interaction between iron and the immune response. 

#### 5.3.1. Investigation of the Effect of Iron on Mycobacterial Growth in Mice

In mice, experimental iron overload can be generated either via the enteral route using an iron-enriched diet or drinking water or via the intraperitoneal route, which is typically accomplished by injecting single or multiple dose of iron-complex. In wild-type mice that received multiple iron-dextran injections (10 injections at 1.2 mg per mouse), *M. avium* (intravenous route 10^6^ CFU) growth was increased in the livers, lungs, and spleens of mice [133]. Similarly, iron-loading mice via polymaltose ferric hydroxide injections (6 injections at 1.25 mg per mouse) allowed for significantly enhanced bacterial burden of virulent *M. tuberculosis* (intravenous route 7.2 × 10^3^ CFU) in the lungs and spleens of mice compared to mice infected without iron loading [142]. The parenteral administration of a high iron dose increases serum iron levels, transferrin saturation and tissue iron deposits. Therefore, circulating iron provides high levels of iron for mycobacterial growth, and the bacteriostatic effect of hyposideremia induced during the first step of infection may be compromised in the presence of high circulating iron. In addition, tissue iron deposition may be toxic and detrimental to the host impacting thus, the host ability to respond efficiently to infection. 

Iron deprivation induced by a low iron diet (6.7 mg/kg for 2 weeks before infection) restricts the growth of *M. avium* (intravenous route infection at 10^6^ CFU) in the livers, spleens and lungs of immunocompetent mice and immunodeficient beige mice [128]. However, mice under dietary iron restriction or severe iron restriction (2 or 6 weeks at 2–6 ppm iron respectively) prior to *M. tuberculosis* aerosol infection does not reproducibly affect mycobacterial growth in the lungs and spleens of mice [111]. Moreover, iron restriction induced by intraperitoneal administration of chelators DFO or HBED in wild-type mice shows small effect on the inhibitory activity of *M. avium* in mice, possibly because chelators have little impact on the iron status [128]. Similarly, extracellular iron depletion by intranasal administration of lactoferrin (1 mg/mL twice a week) did not alter the *M. tuberculosis* burden in wild type mice 22 days after infection [124]. Compared to wild-type mice, lipocalin 2-deficient mice are highly susceptible to intratracheal *M. tuberculosis* infection, with increased bacterial growth observed in alveolar epithelial cells indicating that lipocalin 2 efficiently reduces iron level and inhibits the growth of mycobacteria [120]. 

An experimental iron overload mouse model generated by iron administration *via* drinking water (25 mg/mL) and aerosol infection with *M. tuberculosis* has been shown to exacerbate mycobacterium replication in tissues compared with infected control mice [124]. However, aerosol infection with *M. tuberculosis* in mice after intraperitoneal iron dextran injection (20 mg), which causes high serum iron levels and iron loading in parenchymal cells and macrophages, did not exhibit significant differences in bacterial burdens in the lungs and spleens of mice [143]. Furthermore, mice fed with a mild iron-loaded diet over a long period of time, showed an increased transferrin saturation but mild tissue iron deposits in hepatocytes, which was associated with a decreased mycobacterial burden in liver after an intravenously infection with *M. bovis* BCG compared to uninfected mice [112]. Therefore, the iron distribution and the level of iron overload, as well as the route of iron administration, may yield contradictory results. For future therapeutic design, prospective studies are required to separate the early and long-term effects of circulating iron increase and tissue iron increase on mycobacteria virulence.

The lack of hepcidin which exhibited severe iron overload did not influence significantly *M. tuberculosis* growth in vivo although, in wild-type mice, a decrease in hepatic *hepcidin* mRNA levels was observed after *M. tuberculosis* aerosol infection [111]. Conversely, an upregulation of *hepcidin* gene expression was observed after an intravenous *M. bovis* BCG infection [112], indicating that the expression of hepcidin may depend on the route of infection, the level of mycobacteria inoculation and the virulence of mycobacteria. Eventually, it was shown that hepcidin is selectively protective against siderophilic extracellular pathogens but has no effect on intracellular pathogens [143]. 

The *HFE* gene encodes the α chain of the MHC class-I like molecule that associates with the β-2-microglobulin chain, and *HFE* gene defects are the primary cause of hereditary iron overload (hemochromatosis) in humans [99]. Due to the lack of hepcidin expression, *Hfe*-deficient mice are characterized by iron deposit, notably in hepatocytes, but low iron levels in monocytes was reported [95,144,145]. A more severe phenotype of iron loading is observed in *β-2-microglobulin*-knockout mice [146]. Both *Hfe* and *β-2-microglobulin* knockout mice exacerbated mycobacterial growth after infection compared to wild-type mice, although *Hfe* deficient mice exhibited lower bacterial loads than *β-2m* KO mice [124,146]. In addition, MHC-class I-knockout mice are far less susceptible to *M. tuberculosis* than *β-2-microglobulin*-knockout mice [147], and the depletion of CD8^+^ T cells does not affect the susceptibility of *Hfe*-knockout mice to *M. avium* infection [146]. Furthermore, *β-2-microglobulin*-knockout mice treated with lactoferrin have decreased bacterial loads after mycobacterial infection compared to wild-type and MHC-I-knockout mice [124]. The hypothesis that iron overload increases the risk of active tuberculosis susceptibility is in contradiction with the results obtained using hepcidin knockout mice in mouse model of *M. tuberculosis* infection [111,143].

The modulation of the T cell immune response plays a pivotal role in mycobacterial growth and virulence. The activation of lymphocytes T CD4^+^ Th1-polarised cells is important for the control of intracellular mycobacteria, although CD8^+^ cytotoxic T cells also play a role in the immunity against mycobacteria. A recent study showed that infected mice with *M. bovis* BCG and fed moderately with enriched iron diet exhibited enhanced CD8^+^-T cell recruitment to granulomas [112]. In this model, despite an increase of iron levels, also present in macrophages unlike the hepcidin knockout mouse model, the mycobacterial burden is affected. Thus, specific localization of iron in tissues may be crucial to cell defense activities preventing the growth of mycobacteria in vivo.

#### 5.3.2. Investigation of the Effect of Iron on Mycobacterial Growth in Human 

In humans, several reports have highlighted the influence of iron levels in the body on the outcome of *M. tuberculosis* infection. Iron overload or deficiency can have various causes in human populations, and conflicting observations have been reported. Most of these reports have come from African populations in which tuberculosis is highly prevalent. Studies of several cohorts revealed that high iron levels in the body correlate with an increase of *M. tuberculosis* pathogenicity. 

Iron overload resulting from an increased dietary intake is common in sub-Saharan African adults in rural populations due to the intake of traditional fermented beverage with a high iron content. This affliction is characterized by prominent iron depositions in both macrophages and hepatic parenchymal cells, which is different to the parenchymal iron loading that is predominantly observed in individuals with *HFE* hemochromatosis. The iron overload disorder in the sub-Saharan African population is associated with a poor outcome in patients with tuberculosis [148,149]. In addition, from postmortem analyses of adults from southern Africa, splenic iron overload was significantly associated with death from tuberculosis, with hepatic iron levels having a lower association, suggesting that excess iron may impair the cytotoxic activity of macrophage [150]. 

Tuberculosis is a common opportunistic coinfection in human immunodeficiency virus (HIV)-infected patients, especially in sub-Sahara African countries where HIV is highly prevalent. A cohort study of uninfected or HIV-infected individuals from Zimbabwe, where dietary iron overload is prevalent revealed that increased dietary iron with increased serum ferritin concentrations is associated with a 3.5-fold increase in the estimated odds of developing active tuberculosis after adjusting for HIV status while HIV seropositivity is associated with a 17.3-fold increases risk [151]. The impaired function of T-cells in HIV-infected patients and the combination with dietary iron overload increase the risk for developing virulent tuberculosis. Therefore, it appears that decreasing the prevalence of dietary iron overload in African populations would be beneficial. However, a retrospective analysis from an HIV-infected human cohort from Gambia revealed a significantly greater risk of developing tuberculosis in patients having lower serum transferrin, iron, and hemoglobin, and higher levels of ferritin and hepcidin [152]. In these patients, the elevated hepcidin levels and iron distribution may result from chronic infectious and inflammatory conditions or dietary iron insufficiency, which are both widespread in sub-Saharan African populations and considered as an important contributing factor to anemia [153,154]. Iron supplementation in adult males with pulmonary tuberculosis associated with mild to moderate anemia improves serum iron markers and accelerates the resumption of hematopoiesis in the initial phases of treatment, although it has no influence in the growth of mycobacteria or the clinical outcome of tuberculosis [154]. Therefore, iron supplementation to treat iron deficiency anemia in malnutrition populations would unlikely increase the risk of developing tuberculosis.

Iron deficiency may restrict mycobacterial proliferation but be detrimental for the host. Nutritional iron deficiency is the primary risk factor for developing iron deficiency, with or without concomitant anemia and is highly prevalent in most developing countries but often obscured by infections and inflammatory disorders that are common in the same populations. A study of a cohort from Tanzania suggested that anemia and/or iron deficiency is positively associated with an increased risk of death and tuberculosis recurrence. Indeed, iron deficiency without anemia was observed to be associated with a 2.89-fold increase in the risk of death, while anemia without iron deficiency and iron deficiency anemia was associated with 2.72-and 2.13-fold increased risk of death [155]. Malnutrition, especially when associated with subsequent iron deficiency, modulates immune responses, and affects host defenses against *M. tuberculosis* [156]. Iron is required for proper cell immune functions, and iron deficiency has been shown to compromise cell-mediated immunity. Iron deficiency reduces the number of T-cells and the T cell-induced proliferative response [157,158,159] which can be reversed by the oral or parenteral administration of iron [157,160]. Iron status also modulates cytokine expression profile, leading to immune system impairments and influencing immune response. Indeed, iron-deficient patients show altered activation capability of T-cells and expression of cytokines, with decreased production of IL-6, IL2, IL1, TNFα, IFNγ, and IL-12p40 and increased production of IL-4 and IL-10 compared to cells from healthy individuals [161,162,163]. A recent publication has shown that a mutation in *TFRC* that hinders TfR1-mediated iron internalization, results in defective T and B cell proliferation as well as an impairment of class-switching which is known to be critical for antibody production [164]. Therefore, future therapeutic design should take in consideration that iron modulation influences macrophages polarization and Th1/Th2 cytokine balance. This therapeutic design should also consider the influence of other factors such as the diet, co-infection consequences (co-infection with HIV and malaria), inflammatory disease, other causes of anemia without iron deficiency, and even microbiota which can influence significantly iron absorption [165].

## 6. Conclusions

To combat and eradicate mycobacteria, it is of great importance to tackle the critical paths of a successful infection, including growth, the establishment of mycobacterial dormancy, and mycobacterial escape of immune containment, which determine the outcome of the disease. Iron has either beneficial or detrimental roles in mycobacteria infection, as it is involved in mycobacterial virulence and in the immune cell response of the host. The overall benefit of iron supplementation or deprivation in combating mycobacterial infections may have significant implications for clinical management of individuals at high risk of mycobacterial infection in many developing countries, in coinfection situations, and in iron-deficient or iron overload populations. Conflicting data have been reported on the influence of iron levels on mycobacterial infection outcomes in vivo, which may result from factors affecting iron status, iron distribution and the efficiency of the immune response. 

**Figure 7:**
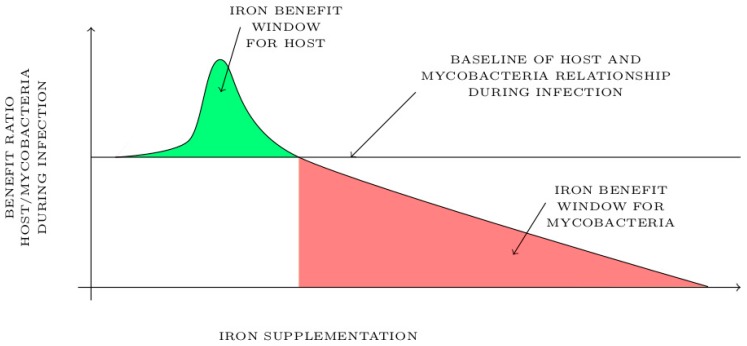
Suggested model of biphasic effect of iron supplementation during mycobacterial infection. A moderate concentration of iron supplementation can have a benefit effect for host during mycobacterial infection (shown in green). However, at high concentration of iron supplementation, iron can be beneficial for mycobacteria promoting growth and virulence (shown in red).

Diet iron supplementation could have a biphasic effect depending on the level of iron status in the host and its distribution in the context of mycobacterial infection. After a thorough literature review, we have identified and modelized in Figure 7 two different effects of iron supplementation on host during mycobacterial infection. As shown in Figure 7, moderate iron supplementation in host could exhibit an “iron benefit window” shown in green during mycobacterial infection. This short window of the beneficial effect of iron supplementation is associated with a moderated reactive oxygen species induction and an increased immune cell response that may attenuate inflammation and mycobacterial burden. Another benefit of this treatment would be the promotion of local hepcidin production, for which anti-mycobacterial proprieties have been described. It is clearly admitted in the literature that a lack of iron may increase the chance of getting an infection, thus it is possible that having a normal–high level of iron could be beneficial for the host in term of preventing mycobacterial infection and having a boosted immune response against infection. Beyond the maximum threshold of the “iron benefit window”, iron supplementation may be detrimental for the host and benefit mycobacteria as shown in red in Figure 7. In this context, iron supplementation promotes bacterial growth and inflammation in response to mycobacterial infection resulting in a poor outcome and a susceptibility of host to infection. 

The future therapeutic use of iron as a pharmaceutical tool to treat or prevent mycobacterial disease will require delineation of the host iron benefit window in the context of infection. Iron treatment should be designed based on the concentration that neither induces host tissue damage as iron overload nor promotes mycobacterial growth. The host iron benefit window should be restrained, and its effectiveness may depend on the level of mycobacterial burden, co-infections and diet habit of host. It might be useful to design personalized co-treatment options against mycobacterial infections such as the combination of iron treatment with traditional drugs used in tuberculosis treatment, such as isoniazid/rifampicin, and determine whether iron status affects the efficacy of the chemotherapy.
